# Impact of Activity-Based Therapy on Respiratory Outcomes in a Medically Complex Child

**DOI:** 10.3390/children8010036

**Published:** 2021-01-09

**Authors:** MacKenzie Goode-Roberts, Scott G. Bickel, Danielle L. Stout, Margaret L. Calvery, Jennifer E. Thompson, Andrea L. Behrman

**Affiliations:** 1 Frazier Rehab Institute, UofL Health, Louisville, KY 40202, USA; mackenzie.goode-roberts@uoflhealth.org (M.G.-R.); danielle.stout@uoflhealth.org (D.L.S.); 2 Department of Pediatrics, University of Louisville, Louisville, KY 40202, USA; scott.bickel@louisville.edu (S.G.B.); Margaret.calvery@louisville.edu (M.L.C.); Jennifer.thompson@louisville.edu (J.E.T.); 3 Department of Neurological Surgery, University of Louisville, Louisville, KY 40202, USA

**Keywords:** spinal cord injury, respiration, trunk control, development, pediatrics, activity-based therapy, cervical injury

## Abstract

Introduction: Activity-based therapies (ABTs) focus on activating the neuromuscular system below the level of spinal cord injury (SCI) promoting neuromuscular capacity. Case description: A 2 year 7 month old with history of prematurity at 29 weeks, neonatal epidural abscess, resultant cervical SCI, respiratory failure, and global developmental delays presented for enrollment in an outpatient activity-based therapy program. Upon presentation to this program, he required nighttime mechanical ventilation via tracheostomy and daytime suctioning. He could not perform any age-appropriate activities and was described by his mother as ‘present’, neither engaged nor attentive. During and after 7 months of participation in ABTs including locomotor training and neuromuscular electrical stimulation, the patient demonstrated unexpected changes in his respiratory status leading to ventilator weaning with concomitant improvements in head and trunk control, participation, development, and quality of life. Discussion: ABT was not only safe for a medically complex child, but also this intervention had a remarkable effect on unresolved respiratory capacity and a more widespread impact on other functions as well as development. A child with a chronic, severe SCI demonstrated positive and impactful improvements in health, functional status, and quality of life during an episode of ABT.

## 1. Introduction

Cervical spinal cord injuries (SCI) often have devastating long-term pulmonary consequences in children [1,2]. Specific manifestations depend on the overall severity of the injury and at what level it occurs [3]. In general, upper cervical cord injuries (C3–C5) result in diaphragmatic paralysis or paresis as well as complete paralysis of intercostal and abdominal muscles [3]. Other complications include the inability to generate effective coughs leading to retained secretions, atelectasis, prolonged illness, and the propensity to infection and pneumonia [3]. These complications are compounded by the seemingly inevitable development of significant scoliosis [1] and restrictive lung disease. Respiratory complications are common in SCI and a leading cause of morbidity and mortality [4]. Children with an upper cervical injury often require permanent tracheostomy with chronic mechanical ventilation [5]. They also have significantly reduced lung function compared to typically developing peers and higher levels of activation of compensatory muscles above the level of the injury [6]. As a result, even in persons who do not require chronic mechanical ventilation, devices to augment secretion clearance, such as those that deliver mechanical insufflation-exsufflation and/or high frequency chest wall oscillation, are often employed with manual therapies or nebulized medications to promote airway clearance and expansion [3].

When SCI occurs in a very premature infant during the neonatal period, chronic lung disease of prematurity and bronchopulmonary dysplasia are often further complicating factors [7]. Children with this constellation of insults are at high risk for needing chronic mechanical ventilation for many years, if not their entire life. We report the case of a two-year-old male with a history of prematurity at 29 weeks gestational age and with an early-onset severe cervical SCI complicated by prolonged mechanical ventilation whose respiratory status improved with intensive activity-based therapy (ABT) [8,9,10].

## 2. Case Presentation

A review of the patient’s medical record revealed that a 29-week gestation male, who initially required continuous positive airway pressure (CPAP) for respiratory support, developed severe lethargy and weakness on approximately day of life 10. A magnetic resonance image of the spine demonstrated an epidural abscess with spinal cord compression from C4 to sacrum. Subsequently, he underwent an epidural abscess drainage and T1–T3 decompression laminectomy. He improved clinically but failed attempts to wean off mechanical ventilation. Thus, tracheostomy along with gastrostomy tube placement was performed at three months of age. At four months of age, he was transferred to an inpatient rehabilitation facility where he received physical, occupational, and speech therapy services. He was then discharged home at seven months old, still requiring mechanical ventilation 24 h/day and with 24-h nursing care. His medical care was guided by a pediatric pulmonologist and home-healthcare. He had three hospitalizations for tracheitis at seven, nine, and nineteen months for nine, sixteen, and nine days, respectively. At one year of age, trials of CPAP with pressure support were initiated when the patient was awake with short periods off of the ventilator, using a cool mist collar. These were generally tolerated well, and he made progress to the point where he used a Passy Muir^®^ speaking valve during the day and was weaning his nighttime rate. He would use 1–2 L per minute (LPM) of supplemental oxygen with viral illnesses.

The patient enrolled in our multi-disciplinary Activity-Based Therapy (ABT) program at 2 years and 7 months of age. His legal guardians signed an Institutional Review Board informed consent (IRB #05.016J) allowing for collection of clinical data and outcomes in a database for retrospective review and dissemination. The parents/caregivers arrived with the patient transporting him using an adaptive stroller, accommodating his ventilator and supplemental oxygen tank. The patient relied fully upon parents/caregivers for transport and mobility.

A comprehensive evaluation at enrollment into the ABT program included medical, physical, and occupational therapy, as well as psychological assessments. On physical examination, he was lying on the exam table in no acute distress, awake and intermittently fussing. His vital signs were temperature 98.8°, heart rate 110 beats per minute (bpm), respiratory rate 60 breaths per minute (bpm), and oxygen saturation 96% in room air. HEENT exam was notable for posterior plagiocephaly, dysmorphic facies, and abnormal dentition with crowding. Chest and cardiac exams were normal. His gastrostomy site was unremarkable. A tracheostomy was in place and was unremarkable. His breathing was unlabored with mild tachypnea and clear and equal breath sounds and was noted to have significant hypotonia throughout his body. The patient utilized the ventilator at night (Vyaire LTV 1150, synchronized intermittent mandatory ventilation (SIMV) with pressure control (PC) 12 cmH_2_O, respiratory rate (RR) 12 breaths per minute, pressure support (PS) 10 cmH_2_O, positive end expiratory pressure (PEEP) 7 cmH_2_O in RA), a Passy Muir^®^ speaking valve at his tracheostomy site during daytime hours, and had continuous heart rate and oxygen saturation monitoring. The patient’s family had end-tidal CO_2_ monitoring available at home as well, which was utilized as needed and generally was in the mid-30s (mmHg) both awake and asleep. Previous physician recommendations included use of high frequency chest wall oscillation to support airway mobilization and clearance with albuterol twice per day and as needed, budesonide nebulized daily, and ciprofloxacin/dexamethasone drops to his tracheostomy twice per day. His caretakers performed airway clearance via tracheal suctioning as required.

**Figure 1:**
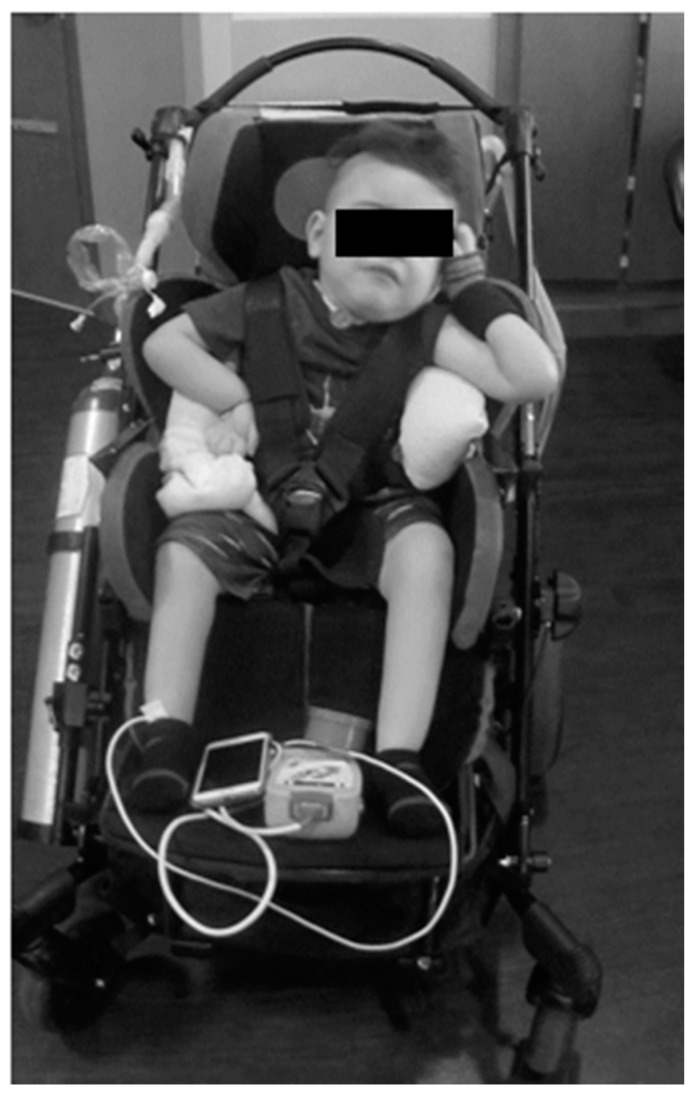
Patient’s initial presentation to clinical program. The patient relies on lateral supports of stroller and additional padding to assist with upright maintenance of trunk; however, he still demonstrates left lateral trunk lean and resting head on head support. He demonstrates preferential posturing with bilateral upper extremities.

The parents reported that home equipment included the adapted stroller, activity chair, ventilator with tracheal suctioning capacity, chest percussion vest, supplemental oxygen, and ankle foot orthoses. His neurologic exam was significant for hypotonia from his chest down to his lower extremities. He had intermittent purposeful movements of the head (side to side), his left arm more than his right arm, and no voluntary movement of his legs. Specifically, his left shoulder posture was flexed and abducted, elbow flexed and supinated, and a flaccid wrist/hand. His right shoulder was subluxed and maintained in internal rotation with elbow flexion and a flaccid wrist/hand. See Figure 1.

With his left arm he initiated elbow flexion, shoulder flexion, and abduction. With his right arm, he only initiated shoulder flexion. No voluntary movement was noted in his bilateral wrists and fingers. He had muscle atrophy in his hands. While arm movements were observed, they were not purposeful for engagement with toys or other items, but at times appeared to meet his laterally flexed head. The patient’s sitting posture was dominated by a severe scoliotic curvature of the spine with concavity to the left with his ribcage protruding and anteriorly rotated. In sitting, he also demonstrated a pelvic obliquity with his left hemipelvis being elevated compared to the right. He was unable to maintain his head in midline often resting to the left. Even during attempts to control, his head would often fall backwards. He was unable to sit upright against gravity, with or without upper extremity support. The Segmental Assessment of Trunk Control (SATCo) [11,12] was scored as a 0/20, indicating that he was unable to maintain his head in midline for 5 s during static sitting with full external support from shoulder girdle to his pelvis. No voluntary movement was observed in his bilateral lower extremities. Patella and Achilles tendon reflexes were absent; ankle clonus and Babinski responses also were absent. The patient was unable to perform any age-appropriate activities: control his head, roll, come to sit, sit, stand, crawl, or walk.

At initial evaluation, the child did not consistently alert to novel items with visual and auditory stimuli, people, and/or surroundings. His affect was blunted, without the use of facial gestures or the expression of emotions to indicate wants, thoughts, and/or needs. He presented without eye contact for name, and without the use of eye contact to mediate and/or deepen interaction. He neither attended to or complied with simple one step commands nor did he use sounds or words to indicate interests or needs. The patient’s only means of communication was via crying, without clear precipitant, with repeated vowel sounds noted without clear intent. Bayley-III assessment [13] revealed non-verbal cognitive skills assessed at the 16-day developmental level, and social and emotional skills fell at the 0 to 3-month level of development. Upon initial evaluation, the mother described her son as “starting to kind of vocalize. He could kind of hold his head up, but you really needed to help support him. He was breathing. He is present”.

Therapy goals were established focusing on improving head and trunk control for sitting, trunk posture, upper extremity engagement, and response to one step instructions. We hypothesized that if his primary therapy objectives were met, his respiratory function would improve in parallel. No targeted respiratory training was planned or initiated. Specific respiratory goals included weaning of invasive ventilator support, less need for suctioning, and ability to tolerate viral infections without significant increases in respiratory support or medications (including hospital admission and use of systemic steroids). Ultimately, the family desired to see the patient reach a point not only where he was free of the need for mechanical ventilation but also where he could safely be decannulated. The family reported, however, that they had been informed that achieving this goal was likely not possible.

### Intervention

The patient was seen for 144 sessions of Activity-Based Locomotor Training (AB-LT) and 90 sessions of Activity-Based Neuromuscular Electrical Stimulation (AB-NMES) over a seven-month period. AB-LT [8,9] is usually provided for 1.5 h/day, 5 days per week, and is conducted in three environments: 1. Treadmill, 2. Overground, and 3. Home/Community. Primary retraining of the neuromuscular system takes place in the treadmill environment for approximately one hour. The patient’s body is supported via a partial body-weight support harness over a treadmill. Trainers provide specific tactile cues to promote kinematic specific standing and stepping intending to activate the neuromuscular system below the lesion. Overground for approximately 30 min, therapists focus on utilizing the activated system during sitting, standing, or stepping. Therapists provide caregiver education during this time to encourage implementation of recovery-based principles [8] into the patient’s home and community.

Due to the patient’s respiratory status, oxygen saturation and heart rate were continually monitored via a toe cuff. Standing time on the treadmill was often dominated by the need for tracheal suctioning as opposed to a therapeutic activity. When oxygen saturation was at or below 92%, per pediatric pulmonologist recommendation, or when the patient demonstrated difficulty managing secretions (i.e., cough, gag, secretions around tracheostomy site), tracheal suctioning took place. Implementation of tracheal capping took place starting at session 93 of AB-LT and session 40 of AB-NMES under direction of the pediatric pulmonologist.

**Figure 2:**
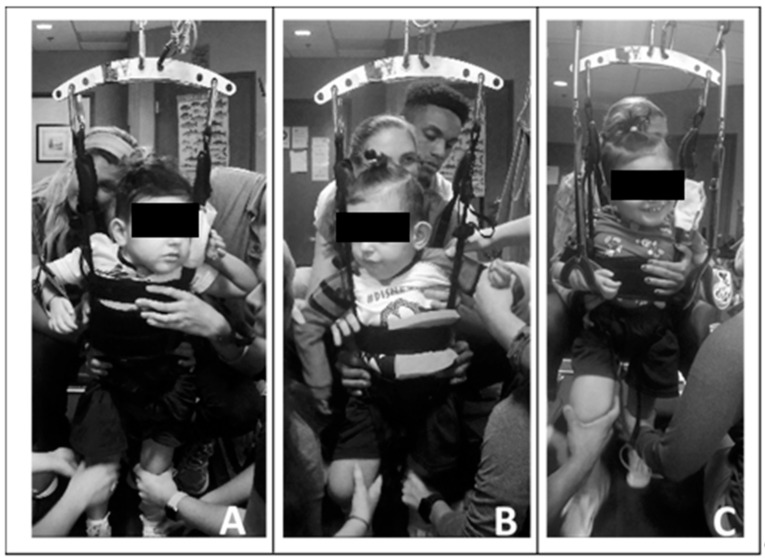
Progress in head and trunk control and engagement across training sessions. (**A**). Session 5, note patient’s use of upright harness strap for a head support. Horizontal trunk strap at height of axillae with manual facilitation providing trunk support. Limited engagement with toys/people. (**B**). Session 24, lowered height of horizontal trunk strap and support, upright and centered head control with visual attention. (**C**). Session 61, consistent upright head and upper thoracic control and initiating activities with his left upper extremity. Engaging with trainers and toys.

Due to his limited head control, initially one trainer primarily assisted with alignment of the patient’s head over his trunk and shoulders and encouraged active midline control. Without manual support, the patient found the vertical support straps of the harness beneficial for holding his head upright. Following the first 20 treadmill sessions, he demonstrated improved ability to attain and maintain his head control independently, and subsequently, the trainer was removed. Utilizing a harness that allowed for variable circumferential support ranging from axillae to pelvis (Power NeuroRecovery, Inc., Louisville, KY, USA) and with manual facilitation, we facilitated a more midline position of the trunk. Due to his scoliosis and limited trunk control, the patient required full trunk support starting at his axillae. As his head and trunk control improved, the harness support was reduced and moved inferiorly, and external manual facilitation was graded accordingly. See Figure 2.

Arm slings were also used in the treadmill environment for support and alignment and allowed for active initiation of purposeful movement and arm swing. In this permissive setting and with gains in head, trunk, and arm control, the therapists integrated play targeting developmentally appropriate activities, e.g., promoting an understanding of cause and effect, with therapeutic tasks.

During the first week of treatment, the patient was diagnosed with a tibial fracture of unknown cause in the setting of osteopenia. He was evaluated and treated with casting by a pediatric orthopedic surgeon. He was restricted from weight-bearing activities for one week and resumed weight-bearing with his cast the following week. During this time, AB-NMES was delivered for trunk and upper extremity control in sitting and standing. Therapy transitioned back to AB-LT once the patient was cleared by the pediatric orthopedic surgeon.

For the first fifteen treadmill sessions, stepping was performed at slower than age-appropriate speeds ranging from 0.3–0.9 miles per hour (mph). Following, all stepping bouts were completed at age-appropriate speeds of ≥1.0 mph. The length of the first 5 treatment sessions was reduced from 90 to 75–85 min to guarantee engagement and participation secondary to drowsiness and fatigue. As tolerance and endurance improved, the session duration quickly extended to 90 min for a full co-treatment of occupational and physical therapies (See Supplemental video). At session 54, physical and occupational therapies split treatment, and he would participate daily in 90 min of AB-LT followed by 60 min of AB-NMES.

AB-NMES was delivered utilizing a customized Xcite (Restorative Therapies, Inc., Baltimore, MD, USA) program to the patient’s bilateral upper extremities and trunk while being supported in a standing frame. Twelve channels, six muscles of the trunk, and upper extremity (left and right), were stimulated at a pulse width of 1000 µs and a frequency of 100 Hz to evoke contractions at the spinal level by stimulating sensorimotor pathways [14,15,16]. Trunk and upper extremity muscles were activated in the context of therapeutic play with manual facilitation guiding movement. As activation improved, therapists decreased the extent of manual facilitation. (See Supplemental video).

**Figure 3:**
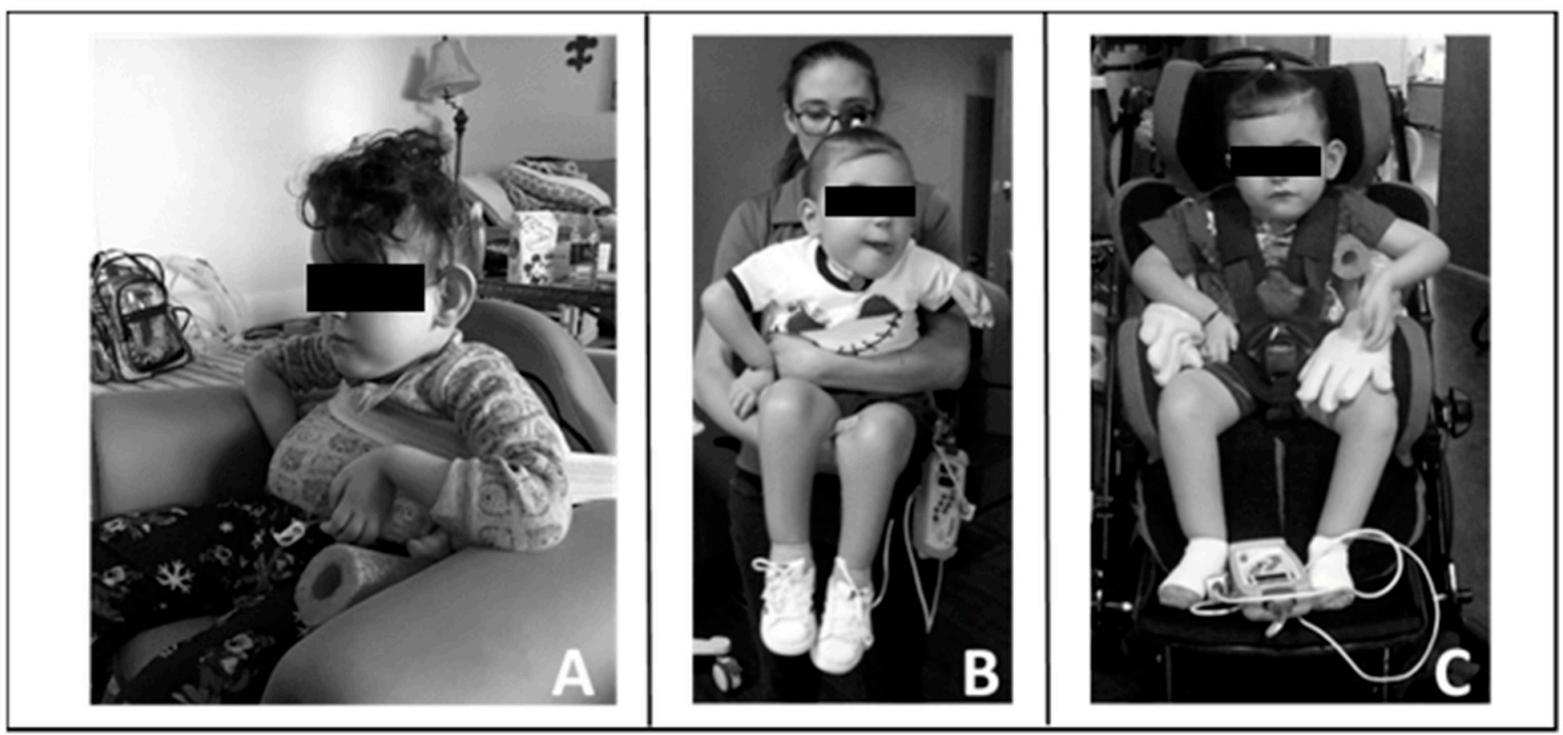
Carry-over of training principles into home and community. (**A**). Modified seat to provide patient with pelvic, lateral, and trunk support. (**B**). Modified carrying position with patient upright allowing for engagement, visual tracking, and head control. (**C**). Modified stroller by bringing patient more upright and added lateral supports to achieve mid-line posture and allow visual engagement and participation.

Throughout his episode of care, the therapists taught the caregivers strategies that reinforced progression and gains in neuromuscular capacity allowing for increased practice in the home and community. Figure 3 provides examples.

## 3. Results

### 3.1. Respiratory Outcomes

**Figure 4:**
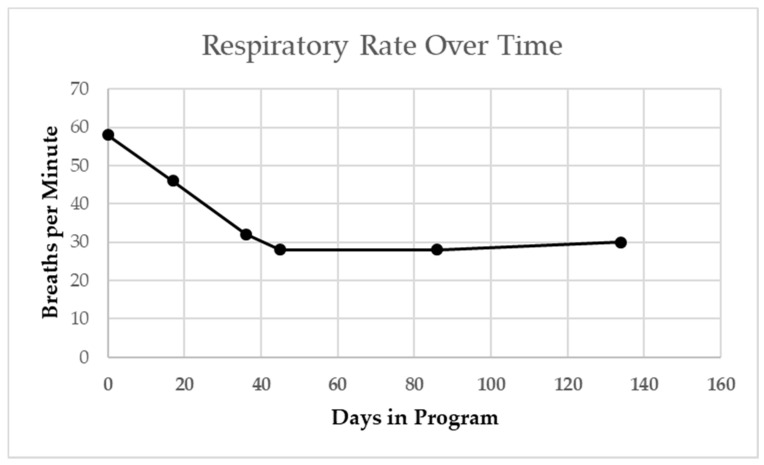
Patient’s resting respiratory rate at primary care and pulmonologist visits. Steady decline from start of activity-based therapy intervention overtime, including when sick with viral cold. Winter season indicated by gray shading. Dashed line indicates age-based median norm.

The patient’s resting respiratory rate as measured at both primary care and pulmonology office visits steadily declined over a 4-month period from 60 to 30 BPM (Figure 4 [17]).

**Figure 5:**
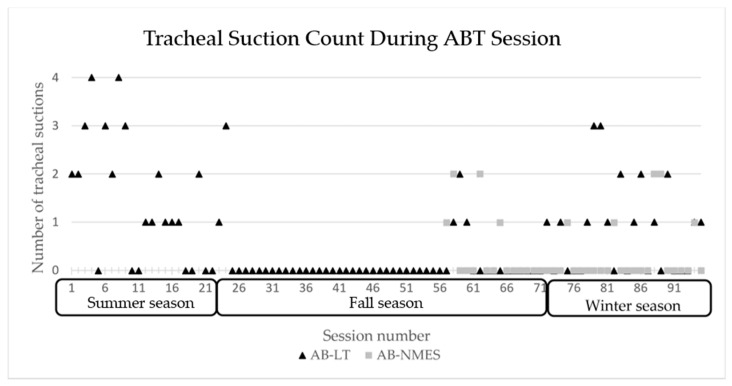
Number of tracheal suctions required during each ABT session 1–94 across seasons. AB-NMES initiated at session number 56. ABT= activity-based therapy, AB-LT = activity-based locomotor training. AB-NMES = activity-based neuromuscular electrical stimulation.

At session 18, he transitioned from using a rate at night to CPAP with pressure support. CPAP was weaned from 7 cmH_2_O to 5 cmH_2_O and well tolerated. He began capping trials during the day and his family reported notable improvements in his respiratory status with time. He required less suctioning and tolerated several viral respiratory illnesses quite well, without need for emergency department visits, admission, medications, or even systemic steroids. The number of times the patient required to be suctioned during therapy sessions declined overall with the exception of periods of viral respiratory illness, see Figure 5.

**Figure 6:**
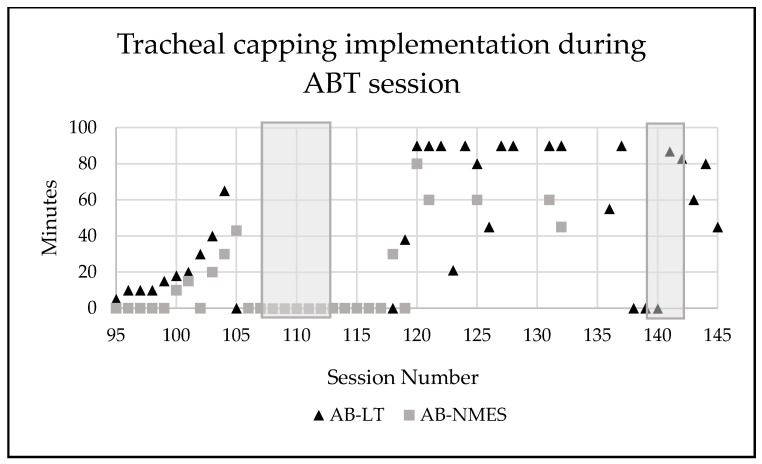
Number of minutes the patient’s tracheostomy was capped during activity-based therapy sessions. Grey shadow indicates a time period where patient was sick (cold, viral infection). AB-LT = activity-based locomotor training. AB-NMES = activity-based neuromuscular electrical stimulation.

Incremental application of tracheal capping took place starting at session 94 until he was tolerating entire AB-LT and AB-NMES treatment sessions consecutively (2.5 h) with maintenance of vitals, endurance, and participation (Figure 6).

After the patient tolerated tracheal capping throughout the day and minimal CPAP with PS settings, he underwent overnight observation to ensure he could tolerate overnight capping. Next, he underwent a polysomnogram where his average oxygen saturation ranged from 94–95% in RA. Mean transcutaneous CO_2_ was 42 mmgHg when asleep (Table 1).

He was noted to have mild to moderate obstructive sleep apnea (apnea hypopnea index 5.9 events/hour when capped, 4.1 events/hour uncapped). As such, he was evaluated by a pediatric otolaryngologist who recommended tonsillectomy and adenoidectomy, and later decannulation. Historically, a cold for this child led to a medical visit, increased use of supplemental oxygen, and the possibility of hospitalization. Across the episode of ABT, the child’s response to viral infections, i.e., a cold, were demonstrably less disruptive to his daily life and he did not require additional medical care. This more typical response to a simple cold had a significant impact on not only the child’s quality of life but also the caregiver’s quality of life.

### 3.2. Trunk Control Outcomes

The patient’s SATCo score increased from 0/20 to 5/20. Thus, he progressed from complete lack of head control to being able to maintain his head and upper trunk with appropriate alignment with support at his axillae during static (head in place for 5 s), active (maintenance of head and trunk control above support during head rotations to the left and right), and reactive testing (appropriate response to perturbation to trunk in each of all four directions).

### 3.3. Developmental Outcomes

While not the direct focus of the ABT interventions, rapid and significant developmental changes were observed by providers, his family, and assessed via the Bayley-III [13]. Marked changes in engagement and social responsiveness were noted. The patient was described as engaging therapy staff with his facial expressions and eyes, engaging in reciprocal maintained non-verbal communication. At the same time, vocalizations increased and differentiated. His family reported that his crying became increasingly specific to need with repeated use of sounds and several spontaneous words noted. From a motor standpoint, the patient acquired the ability to visually track people and toys, with use of eye contact to communicate yes and no. The patient was able to bring his left upper extremity to toys and a tabletop to participate in activities without physical assistance. The therapists and his family noted that he was as able to understand simple cause and effect. Bayley-III assessment at discharge revealed dramatic developmental changes; non-verbal cognitive abilities improved from that of a 16-day old to a 9-month developmental level and social/emotional skills from 0–3 month to 15–18-month developmental level.

At discharge, the parents could describe their child’s emerging personality traits, ‘stubborn, willful, playful’ and anticipated that ‘he can have a normal life now, we can have a normal life’. He was able to engage in play with his family, e.g., listening intently to stories, assisting in turning book pages, wanting to pet the dog, and knocking toy trucks off a table. The child is scheduled for routine follow-up every 6 months–1 year.

## 4. Discussion

A history of a two-year period of mechanical ventilation, a high cervical injury, and premature birth all led to a poor prognosis for achievement of independent breathing by this patient. Current standards of medical care and rehabilitation provided little expectation of a change in any outcomes due to rehabilitation for this child with a complex medical and chronic condition of tetraplegia. During a course of ABTs, this child exhibited remarkable progress in his respiratory capacity reducing his ventilator dependency and improving his respiratory health. Furthermore, he developed head and upper trunk control, as well as demonstrated gains in multiple domains of development, e.g., social, cognitive, behavioral. Meaningful improvements in the quality of life of the child and family were observed. Exposure to an intensive episode of care employing ABTs was associated with the foremost, unexpected change in respiratory capacity and other physical and developmental gains.

A plethora of basic science evidence supports the notion of a strong, neural coupling between respiratory and locomotor networks [18,19,20]. Synergy between the onset and continuation of locomotion (i.e., centrally generated stepping) with increased respiratory rhythmicity may provide a rationale for the improved respiratory capacity of this patient in association with intense, activity-based locomotor training. In contrast to direct, intense respiratory training [21,22] specifically targeting respiratory challenge, this patient was assisted via overhead partial body weight support with manual facilitation of therapists and trainers to both stand and step for one hour per day, 5x/week on a treadmill followed by activities to promote upright trunk control in sitting and standing in the clinic and home. In response to this postural change and activity, his respiratory capacity remarkably improved noted by decreased respiratory rate, decreased number of times suctioning necessary per therapy session, reduced reliance on mechanical ventilation with capping of tracheostomy, improved response to a typical cold, and being deemed a candidate for decannulation following tonsillectomy and adenoidectomy.

In adults with chronic SCI, a mean of 62 ABT sessions improved pulmonary function outcomes, measured by forced vital capacity, forced expiratory volume in one second, and maximum inspiratory and expiratory pressures [23]. Hormigo, et al. [24] postulated that ABT and related therapies such as neural and muscular stimulation, which promote neuroplasticity and activation of neural circuitry below the lesion, may have beneficial respiratory effects. While tightly linked neural connections between respiratory and locomotor networks may contribute to the effects of ABT, other possible mechanisms include establishment of new neural pathways, activation of previously silenced respiratory muscles, improved activation of compensatory muscles, and improved trunk/positional control as a result of therapy.

The patient in this case improved in multiple respiratory domains, which resulted in a significantly improved quality of life by the family’s report and placed him on a clear path to decannulation, previously an outcome the family had been told would likely never happen. The management of a child who is dependent on a ventilator requires intermittent suctioning throughout the day, and continual monitoring of vital signs is conducted by vigilant caregivers in a context of anxiousness for their child’s health and well-being [25]. Even the improved response of the child to a simple cold significantly altered the quality of life of child and caregiver.

The patient’s improved respiratory trajectory as well as gains in trunk control are similar to that of another child on whom we recently reported [10], though that patient had not required the use of chronic mechanical ventilation. The patient in this case report had never developed head control, and following intervention, he gained both head and upper thoracic trunk control. The improvement in head and trunk control via therapy may be one contributing mechanism for the gains seen in respiratory capacity. Importantly, our patient tolerated therapy quite well, with no adverse side effects or transient worsening of respiratory status, implying that, with close monitoring and care coordination, ABT is both safe and effective even for medically complex children requiring chronic mechanical ventilation.

While not the focus of the ABT intervention, marked and unforeseen changes were observed across developmental domains including social-emotional and non-verbal/cognitive. Developmental gains were noted across environments, sustained, and self-initiated. Evidence from the field of development may provide insight into factors contributing to these changes. During ABT, the patient’s orientation was dramatically changed by bringing him into an upright standing, weight-bearing position and during facilitated stepping. This was in strong contrast to his daily and constant experience of being reclined, whether stationary or during passive transport. The upright and locomotor experience of ABT may have provided an environment conducive to activation and development of the visual, vestibular, and somatosensory systems [26,27,28]. Additionally, some of the developmental, cognitive improvements of this patient may be explained by the nature of ABT where exercise (therapies) takes place in a cognitively-engaging and enriched environment [26].

Not only are ABTs safe for medically complex children, but this intervention may also have a more widespread impact on other physiologic [23,29] systems as well as psychological development. Medical referral should be considered for children with complex, medical history of SCI and respiratory compromise to intensive ABT programs. With a high risk for secondary complications [1] when injured less than 5 years of age and increased risk for developing scoliosis the younger the age of injury [30], ABTs may effectively mitigate the consequences of paralysis, immobility, and early sensory deprivation. This child’s medical history and response to ABT supports this comprehensive effect on the child’s health, functional status, and quality of life and it has the potential for benefit to other pediatric patients with early-onset SCI.

## 5. Conclusions

In a child with a complex medical history including cervical spinal cord injury and respiratory compromise, ABTs: locomotor training and neuromuscular electrical stimulation, were safe and feasible in an out-patient therapy program. Unexpected improvements in respiratory function and development achieved by this patient during an intense episode of ABT provides justification for referring children with such complex medical needs to programs delivering ABT interventions.

## Figures and Tables

**Table 1:** Sleep study results. TST = total sleep time, AHI = apnea-hypopnea index, REM = rapid eye movement, OAI = obstructive apnea index, OHI = obstructive hypopnea index, CAI = central apnea index, h = hour, SpO_2_ = peripheral oxygen saturation.

Tracheostomy	Time	TST (min)	AHI (/h)	REM AHI (/h)	OAI (/h)	OHI (/h)	CAI (/h)	Oxygen Nadir	SpO_2_ Avg during Sleep
Capped	9:39:07 P.M.–1:09:07 A.M.	203.0	5.9	10.0	0.0	4.4	1.5	83%	94%
Uncapped	1:09:07 A.M.–6:06:25 A.M.	290.8	4.1	9.9	0.0	3.5	0.4	86%	96%

## Data Availability

The data presented in this study are available on request from the corresponding author. The data are not publicly available as this is a clinical case report.

## Supplementary Materials

The following are available online at https://www.mdpi.com/2227-9067/8/1/36/s1, Video S1: Activity-based therapy interventions.
